# Irisin and exercise training in humans – Results from a randomized controlled training trial

**DOI:** 10.1186/1741-7015-11-235

**Published:** 2013-11-05

**Authors:** Anne Hecksteden, Melissa Wegmann, Anke Steffen, Jochen Kraushaar, Arne Morsch, Sandra Ruppenthal, Lars Kaestner, Tim Meyer

**Affiliations:** 1 Institute of Sports and Preventive Medicine, Saarland University, 66123 Saarbrücken, Germany; 2 Deutsche Hochschule für Prävention und Gesundheitsmanagement, 66123 Saarbrücken, Germany; 3 Institute for Molecular Cell Biology, Saarland University, 66421 Homburg, Germany

**Keywords:** Myokine, Browning, FNDC5

## Abstract

**Background:**

The recent discovery of a new myokine (irisin) potentially involved in health-related training effects has gained great attention, but evidence for a training-induced increase in irisin remains preliminary. Therefore, the present study aimed to determine whether irisin concentration is increased after regular exercise training in humans.

**Methods:**

In a randomized controlled design, two guideline conforming training interventions were studied. Inclusion criteria were age 30 to 60 years, <1 hour/week regular activity, non-smoker, and absence of major diseases. 102 participants could be included in the analysis. Subjects in the training groups exercised 3 times per week for 26 weeks. The minimum compliance was defined at 70%. Aerobic endurance training (AET) consisted of 45 minutes of walking/running at 60% heart rate reserve. Strength endurance training (SET) consisted of 8 machine-based exercises (2 sets of 15 repetitions with 100% of the 20 repetition maximum). Serum irisin concentrations in frozen serum samples were determined in a single blinded measurement immediately after the end of the training study. Physical performance provided positive control for the overall efficacy of training. Differences between groups were tested for significance using analysis of variance. For *post hoc* comparisons with the control group, Dunnett’s test was used.

**Results:**

Maximum performance increased significantly in the training groups compared with controls (controls: ±0.0 ± 0.7 km/h; AET: 1.1 ± 0.6 km/h, *P* < 0.01; SET: +0.5 ± 0.7 km/h, *P* = 0.01). Changes in irisin did not differ between groups (controls: 101 ± 81 ng/ml; AET: 44 ± 93 ng/ml; SET: 60 ± 92 ng/ml; in both cases: *P* = 0.99 (one-tailed testing), 1−β error probability = 0.7). The general upward trend was mainly accounted for by a negative association of irisin concentration with the storage duration of frozen serum samples (*P* < 0.01, β = −0.33). After arithmetically eliminating this confounder, the differences between groups remained non-significant.

**Conclusions:**

A training-induced increase in circulating irisin could not be confirmed, calling into question its proposed involvement in health-related training effects. Because frozen samples are prone to irisin degradation over time, positive results from uncontrolled trials might exclusively reflect the longer storage of samples from initial tests.

**Trial registration:**

Clinicaltrials.gov. Identifier: NCT01263522.

## Background

Although the existence of health-promoting effects of regular physical exercise is beyond doubt, our knowledge about the underlying molecular, cellular, and systemic mechanisms involved in such effects is still fragmentary. In 2012 a new muscle-derived messenger substance (myokine) was described. This newly identified myokine was named ‘irisin’ after the Greek messenger goddess Iris [1], and presumably is an important link within the causal chains leading from physical activity to better health [1]. This finding has received great attention, and resulted in a wealth of comments and secondary publications [2-8]. In their original work, Boström *et al*. [1] unfolded an impressive line of evidence for the health-related benefits of irisin, starting with the observation of improved health and longevity in transgenic mice with mild overexpression of peroxisome proliferator-activated receptor gamma coactivator-1α (PGC-1α) in muscle, which is a model for the well-known induction of PGC-1α by physical exercise. The ensuing experiments led to the characterization of a membrane protein (FNDC5) that is expressed under control of PGC-1α in skeletal muscle. The extracellular part of FNDC5, cleaved and secreted into the extracellular space, causes similar systemic effects to those seen in the mouse model, namely, increased energy expenditure and weight loss, and improved glucose homeostasis.

The initial results [1] were derived mainly from experiments using transgenic animals and cultured cells. Their external validity was substantiated in wild-type mice and humans by a significant, training-induced increase in FNDC5 expression in skeletal muscle and by an increase in the serum concentration of irisin. In humans, a twofold increase in circulating irisin after a training period of 10 weeks was reported; however, given the low subject number (n = 8) and lack of a control group, this result has only a preliminary character. In the meantime, two studies were unable to reproduce a substantial, training-induced increase in FNDC5 expression [9] or circulating irisin [10]. However, randomized controlled trials (RCTs) are still lacking. Therefore, we determined the concentration of irisin in the serum of 102 subjects from a prospective RCT of training. In addition, we assessed the proposed influencing factors of baseline irisin concentration and the respective training-induced changes.

## Methods

### General design

**Figure 1:**
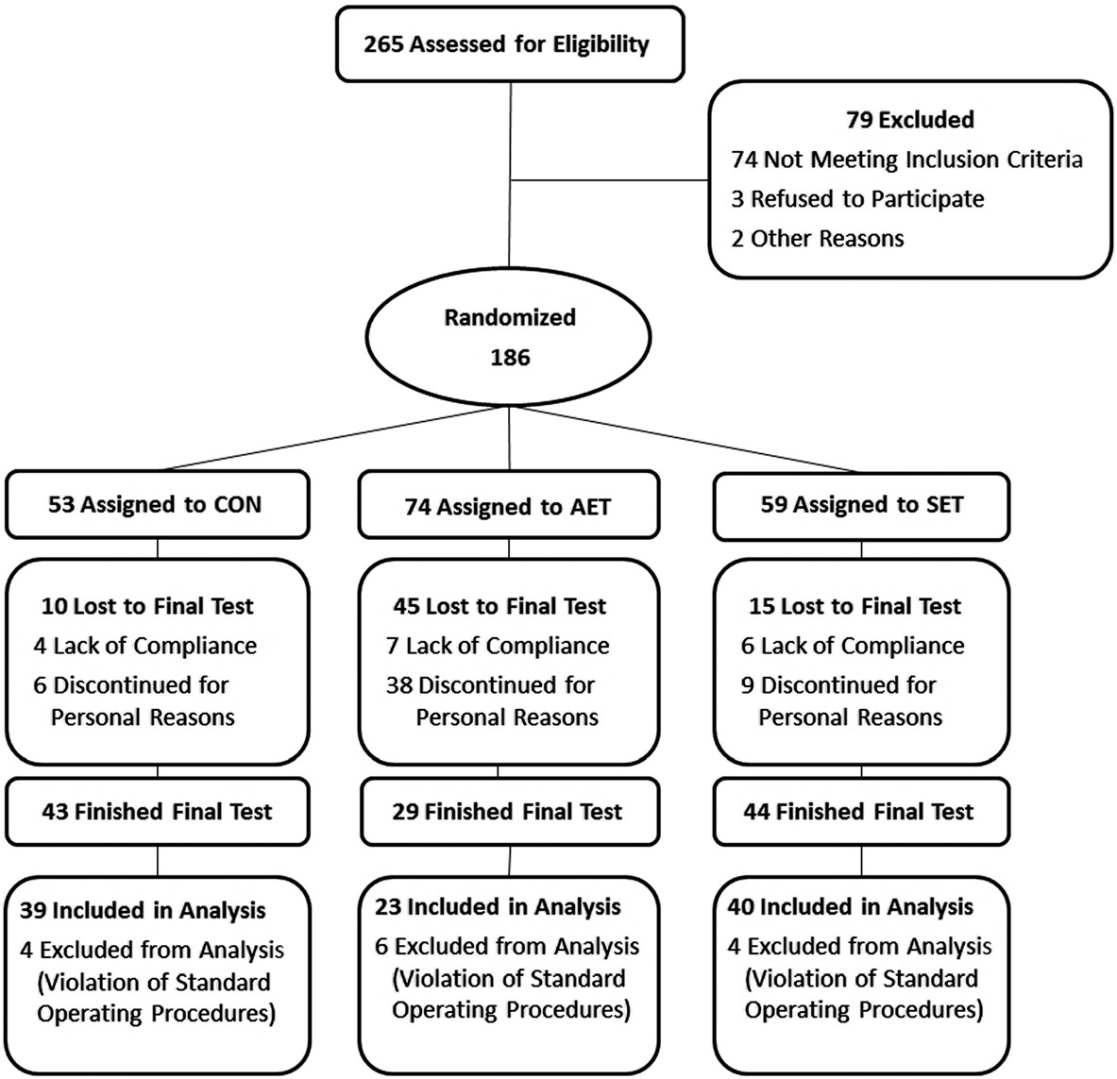
**Trial design.** CON, control group; AET: aerobic endurance training; SET: strength endurance training.

The study was a prospective RCT of training with an intervention period of 6 months. Two guideline-compliant preventive training programs (aerobic endurance training (AET) [11,12] and strength endurance training (SET) [12,13]) were studied in parallel groups (Figure 1). All tests were conducted at Saarland University.

### Ethics approval

The present investigation was carried out in accordance with the declaration of Helsinki and approved by the local ethics committee (Ärztekammer des Saarlandes, ID 148/10). All participants provided written informed consent prior to participation.

### Subjects

Subjects were selected to represent typical healthy participants in preventive training. Inclusion criteria were: age 30 to 60 years, untrained status (<1 hour/week regular activity for at least 1 year), non-smoker, body mass index <30 kg/m^2^, no major diseases or disorders that would interfere with the intended training or require regular medication (including fasting blood glucose concentration <6.1 mmol/l, resting blood pressure <160/100 mmHg, presence of iron deficiency/anemia). Subjects were recruited by advertisements in local media and at informative events. Randomization was performed as simple random allocation; each subject identifier was forwarded to a person who had no further relationship to the conduct of the study, and who performed the randomization blindly using a computer list.

### Serum collection and handling

Testing sessions were scheduled before the training period and at 2 to 7 days after the final training bout. Subjects were instructed to abstain from physical exercise between the final training bout and the final test. Participants reported to the laboratory between 08.00 and 10.00 hours after an overnight fast; the time of day was held constant for every subject (± 30 minutes). Venous blood samples were taken from the antecubital vein after a supine resting period of at least 10 minutes. Blood samples were centrifuged immediately after collection, and serum aliquots were stored frozen at −20°C until analysis. Glucose, insulin, and cholesterol were analyzed by routine techniques (UniCell DxC 600 Synchron; Beckmann Coulter GmbH, Krefeld, Germany).

### Determination of circulating irisin

A commercial ELISA kit [10] (Phoenix Pharmaceuticals, Burlingame, CA, USA) was used to determine the serum concentration of irisin, which was measured by a Sunrise microplate reader (Tecan, Männedorf, Switzerland). The lot-specific protocol supplied by the manufacturer was fully respected. With each 96-well plate, a standard solution with an irisin concentration of 41.0 ng/ml (supplied by hoenix Pharmaceuticals) was measured. The 10 measurements gave a value ± standard deviation (SD) of 41.3 ± 1.8 ng/ml. To minimize differences in the handling of the individual wells of the immunoplates used for this assays, a semi-automatic 96-channel pipettor (Selma, CyBio AG, Jena, Germany) was used. All measurements were performed within one experiment immediately after completion of the training study, and samples were prepared in triplicate. Sample values were determined by extrapolation to a standard curve (determined for each measurement) using the curve-fitting procedure in Matlab (MathWorks, Ismaning, Germany). All samples were coded to ensure blinding of the test operator.

### Anthropometry

Height and weight were measured for all participants while they were wearing light sports clothes without shoes. Body mass index (BMI) was calculated as weight (kg) divided by height squared (m^2^). Body fat percentage was determined with a Harpenden skinfold caliper (Wilken, Berlin, Germany) using the 10-point method [14]. Waist circumference was measured halfway between the lower rib and the iliac crest in a horizontal plane. Hip circumference was measured as the widest horizontal circumference over the buttocks.

### Exercise testing

Exhaustive exercise tests were carried out on a motor-driven treadmill (Woodway ELG 70, Woodway GmbH, Germany) using a combined step-wise and ramp-wise protocol as previously published [15,16]. In brief, an initial step-wise phase (step duration: 3 minutes, step increment: 1 km/h), intended for the determination of submaximal heart rate curves, was followed by a ramp-shaped phase (ramp increment: 0.8 km/h/min) to allow for the valid determination of maximum physical capacity. Initial velocity was chosen as between 3 and 7 km/h, according to subject characteristics (gender, age, BMI). The individual protocol from the initial test (initial velocity and number of steps) was held constant for the final test. Treadmill incline was 0.5 degrees. Maximum heart rate (≥200 minus age (years)) and blood lactate concentration (≥8 mmol/l) were used as indicators of exhaustion, and maximum physical performance was analyzed only in subjects who fulfilled these criteria in both exercise tests. Capillary blood samples for the determination of blood lactate concentrations were taken from the hyperemized left earlobe at rest and 2 minutes after cessation of exercise. Samples were immediately hemolyzed, and analysis was carried out using an enzymatic amperometric system (Super GL, Greiner, Flacht, Germany). The test operators were blinded to subject allocation.

### Interventions

Subjects in the training groups exercised 3 times/week for 26 weeks. The minimum compliance to allow inclusion in the final analyses was defined at 70% (55 training sessions).

Aerobic endurance training (AET) consisted of 45 minutes of walking/running at 60% heart rate reserve. Heart rate monitors with integrated memory function (Polar, Kempele, Finland), programmed with each participant’s personal heart rate prescription (± 5 beats/min) were provided to the participants for control and documentation of training. Participants were required to attend at least one supervised training session per week. At this occasion training and heart rate logs were inspected.

Strength endurance training (SET) consisted of eight machine-based exercises (back extension and crunch; Dr Wolff, Arnsberg, Germany), pulldown, seated row, seated leg curl, seated leg extension, seated chest press, and lying leg press (gym80 International, Gelsenkirchen, Germany). Two sets of 15 repetitions with 100% of the 20 repetition maximum were performed for each exercise. For SET, all training sessions were performed under the supervision of study staff.

Participants in the control group were advised not to change their previous sedentary lifestyle. Regular phone contact ensured compliance.

### Statistical analysis

Distributions of categorical variables were tested by the Kruskal-Wallis test. For continuous variables, normal distribution was checked by the Shapiro-Wilk test. If appropriate, variables were log-transformed for statistical testing. Differences between groups were tested for significance using analysis of variance (ANOVA). For *post hoc* comparisons with the control group Dunnett’s test was used (one-tailed, in accordance with the study hypothesis). Multiple regression analysis was used to analyze influencing factors on baseline irisin levels and training-induced changes, respectively. The two training groups (AET and SET) were pooled for regression analysis of training-induced changes. Effects coding was used for nominal parameters.

**Figure 2:**
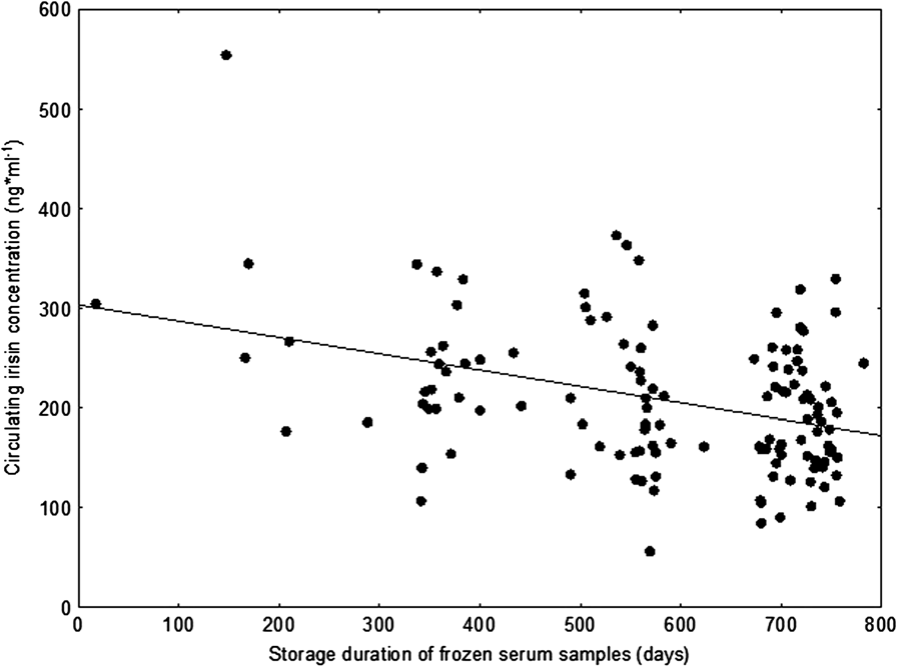
**Association between sample storage duration and serum irisin concentration.** Data points represent baseline values of all groups and final tests of control subjects.

We found an unexpected effect of sample storage duration on irisin concentration, therefore arithmetical correction for this confounding factor was performed. For this additional calculation, baseline values from all groups were pooled with the final tests of control subjects in order to obtain data points with a wide range of storage periods (range 17 to 782 days). Irisin concentrations were plotted against the storage period, and linear regression was performed (Figure 2). Correction of irisin concentrations was based on the slope of the regression line (0.184 ng/ml/day).

The significance level for the α-error was set at *P* < 0.05. Data are presented as means ± SD if not otherwise indicated. The Statistica 7 software package (StatSoft, Hamburg, Germany) was used for data analysis.

## Results

### Baseline subject characteristics

Baseline subject characteristics are summarized in Table 1. There were no significant differences between groups.

**Table 1:** Baseline subject characteristics

Characteristic*	**Control (n = 39)**	**AET (n = 23)**	**SET (n = 40)**	***P***
Participants (female/male), n	39 (26/13)	23 (15/8)	40 (23/17)	0.675
Age, years	50 ± 7	49 ± 7	48 ± 7	0.370
BMI, kg/m^2^	24.5 ± 3.1	23.5 ± 3.5	24.9 ± 3.4	0.253
Body fat, %	24 ± 5	23 ± 4	23 ± 5	0.652
V_max_,, km/h	10.0 ± 1.5	10.3 ± 1.7	10.0 ± 1.4	0.921
Irisin,, ng/ml	188 ± 75	201 ± 52	196 ± 51	0.713
Insulin, mU/l^2^	4.8 ± 2.1	4.4 ± 2.3	5.2 ± 2.8	0.513
Cholesterol, mg/dl^2^	211 ± 38	221 ± 40	206 ± 43	0.375

AET, aerobic endurance training; BMI, body mass index; SET, strength endurance training; V_max_, maximum performance in the exercise test.

^1^Values are mean ± SD unless otherwise stated.

^2^SI conversion factors: to convert insulin to pmol/l, multiply values by 6.945; to convert cholesterol to mmol/L, multiply values by 0.0259.

### Physical performance

**Figure 3:**
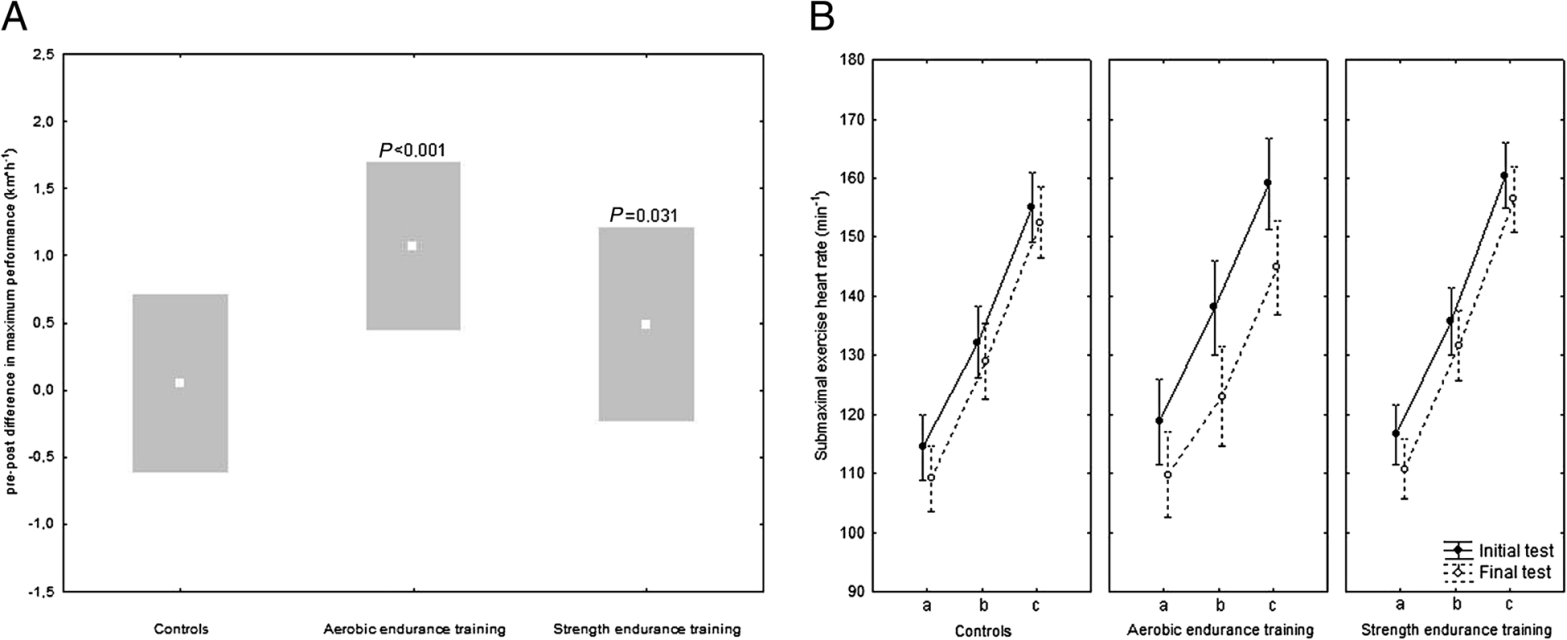
**Changes in physical performance over the intervention period. (A)** Maximum running speed (mean ± standard deviation). *P*-values indicate differences from control group (*post hoc* Scheffé test). **(B)** Courses of submaximal exercise heart rate (means ± standard error). Letters (a-c) on x-axes indicate the final three steps preceding the transgression to the ramp-shaped phase of the exercise tests. Group × test × step interaction: *P* = 0.039.

In total, 95 subjects fulfilled the exhaustion criteria in both exercise tests (Con 36; AET 21; SET 38) and were included in the analysis of maximum performance. Maximum performance in the treadmill test increased significantly in the AET and SET groups compared with the control group (Figure 3A).

Courses of submaximal exercise heart rate during the step-wise phase of the exercise tests are depicted in Figure 3B. The clear downward shift in AET was substantiated by the significant group × test × step interaction (*P* = 0.039).

### Baseline irisin concentration

Mean baseline irisin concentration was 194 ± 62 ng/ml. There were no significant differences between groups (Table 1). Based on a panel of previously described predictors [9,10,17,18] only age (*P* = 0.029, β = −0.20) and total cholesterol (*P* = 0.015, β = 0.20) showed a significant, independent influence on circulating irisin levels. Other, but non-significant, predictors in the model were sex (*P* = 0.089, β = 0.23), BMI (*P* = 0.362, β = 0.13), percentage body fat: (*P* = 0.119, β = −0.22), maximum physical performance (*P* = 0.474, β = −0.08), and insulin level (*P* = 0.068, β = 0.17). However, the strongest predictor was the duration of the storage period of frozen serum samples (*P* < 0.001; β = −0.33). When irisin concentrations were corrected for storage duration using the slope of the regression line (0.184 ng/ml/day) the following values were obtained: overall mean: 307 ± 63; control 302 ± 77; AET 311 ± 56; SET 311 ± 51 ng ± ml (no significant differences between groups).

### Changes in circulating irisin over the intervention period

**Figure 4:**
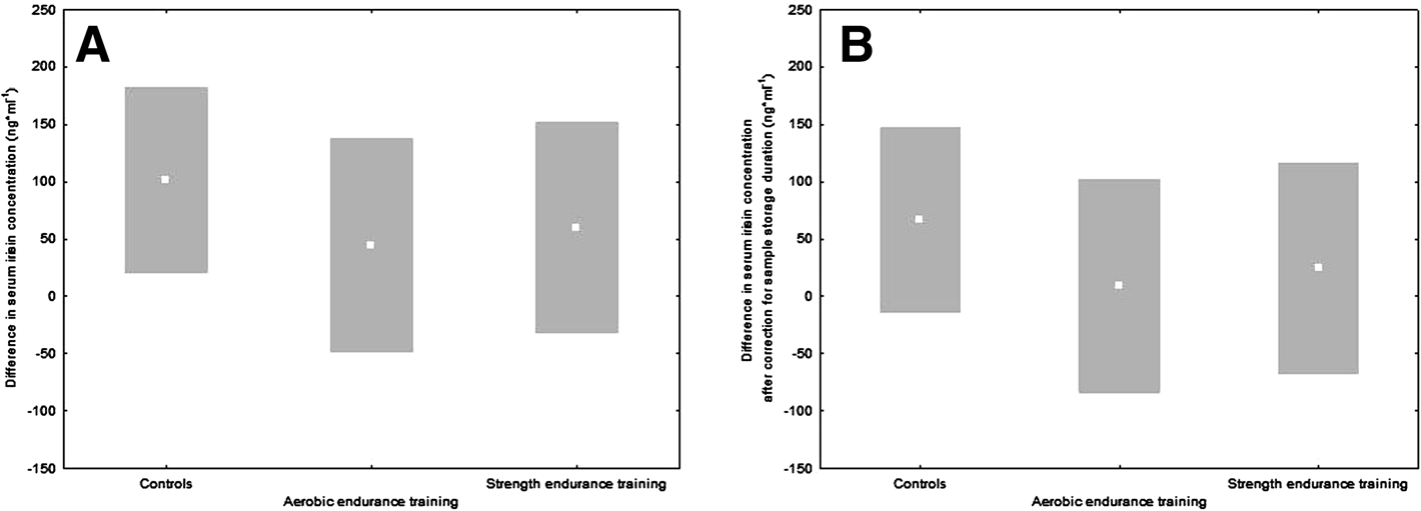
**Changes in serum irisin concentration over the intervention period. (A)** Raw values as measured (mean ± standard deviation). **(B)** Values after correction for the influence of sample storage duration using the slope of the regression line for baseline values (0.184 ng/ml/day; mean ± standard deviation).

Changes in raw values of circulating irisin are displayed in Figure 4. There were no significant differences between groups. (*P* = 0.99 in both cases; 1−β error probability = 0.7). The general upward trend in all groups was mainly accounted for by the longer storage of samples from initial tests. Figure 4 displays the changes after arithmetically correcting for this confounder (*P* = 0.99 in both cases, 1−β = 0.7).

No relation between changes in circulating irisin in the training groups and age, gender, BMI, or baseline irisin concentration was found by multiple regression analysis (age: *P* = 0.911, β = −0.01; gender: *P* = 0.997, β < 0,01; BMI: *P* = 0.353, β = 0.14, baseline irisin: *P* = 0.158, β = −0.18). However, from the individual assessment of outliers (below or above the mean ± 2 standard deviations) it is noteworthy that six of the eight low responders were male, whereas eight of the ten high responders were female.

## Discussion

Irisin has been described as a new muscle-derived messenger substance (myokine), which may be involved in the mediation of systemic, health-related benefits from regular physical exercise. Although the original paper identifying this substance presented a stringent and conclusive sequence of *in vitro* and transgenic animal experiments, the evidence for induction by exercise training in humans remains preliminary [1]. In the present RCT, a training-induced increase in the serum concentration of irisin could not be shown, despite a clear increase in physical performance, which must be considered as a positive control for the overall efficacy of training interventions.

In their original paper, Boström *et al*. [1] reported a twofold increase in circulating irisin after 10 weeks of endurance training in healthy, obese adult humans. However, because of the low subject number (n = 8) and lack of a control group, this result warrants confirmation. Moreover, the statistical procedure used to test the pre-training and post-training difference merits discussion (Student’s *t*-test for the eight subjects after internal normalization for pre-training values resulting in a pre-training variance of 0). In the meantime, two further articles have been published, which failed to substantiate a robust, systematic training-induced increase in the expression of the irisin precursor FNDC5 [9] or in circulating irisin [10]. However, interpretation was complicated by methodological limitations in both cases.

Timmons *et al*. reported gene-chip data from two training trials (6 weeks of endurance training (n = 24) and 20 weeks of whole body strength training (n = 43), respectively) and a cross-sectional study (young and old groups of trained and age-matched sedentary subjects (n = 10 per group)) [9]. A statistically significant difference in FNDC5 mRNA could only be shown between the older endurance-trained and sedentary subjects. Power calculations, which seem crucial in order to interpret statistically insignificant results, were not provided. The interpretation of these results is controversial, particularly when the reported lack of association with differences in metabolic health indicators is considered. The authors [9] concluded that FNDC5 is not systematically induced by exercise and is unlikely to play a major role in health-related training effects. In their reply, Boström *et al*. emphasized the semiquantitative character of gene-chip analyses as a possible explanation for the smaller effect sizes and the lack of correlation with health indicators. However, this was only stated in general without considering the sensitivity data provided in the original work [9]. Boström *et al*. also point out the lack of increase in the expression of PGC-1α, which they considered a crucial positive control of training efficacy on the molecular level. However, although a chronic, training-induced increase in PGC-1α is well established on the protein level, the acute exercise-induced increase in PGC-1α mRNA seems to be a transient phenomenon [19]. In this context, it is also surprising that two [20,21] of four [19-22] studies, referenced to substantiate the value of PGC-1α as a positive control for the efficacy of a prolonged training program, reported on acute changes after a single bout of exercise only. Moreover, an increase in mitochondrial enzyme activities, undoubtedly markers of exercise-induced adaptation in skeletal muscle, was found in the endurance training group by Timmons *et al*. [9,23], a fact that was not considered in the reply by Boström *et al*.

Huh et al. [10] determined circulating irisin in serum samples from a training study first published in 2011. This investigation was originally designed to compare two repeated-sprint protocols [24]. The two training groups were apparently pooled for the retrospective analysis of circulating irisin. A significant acute increase in circulating irisin after a bout of sprints (four or six bouts of 80 m sprints) was found before but not after the 8 week training period. Surprisingly, the chronic training-induced change in resting values, which is a far more relevant marker of the proposed physiological role of irisin, was not tested. Numerically, a slight decrease in irisin was apparent (473.4 ± 36.4 vs. 420.3 ± 38.5 ng/ml); however, the training protocol (four or six bouts of 80 m sprints, 3 times/week) and the subject characteristics (physical education students) differed widely from the original work of Boström *et al*. and are not representative of preventive training.

In the present study, we could not detect any training-induced increase in circulating irisin, despite the proven efficacy of training interventions as documented by physical performance. Moreover, use of previously untrained subjects and a long period of guideline-compliant exercise training must be regarded as favorable for the generation of detectable training effects. Consequently, the data from the eight subjects presented in the original work of Boström *et al*. [1] remain the only evidence for an induction of circulating irisin by exercise training in humans. Moreover, the lack of stability of irisin in frozen serum samples, which was apparent in our data, may lead to an erroneous increase in irisin for intra-group comparisons, because of the shorter storage of samples from the later tests (Figure 4). This issue is particularly relevant because there are no details about the preparation and storage of serum samples or the delay between completion of the training study and analysis in either the original work by Boström *et al*. [1], or the publication of the training trial using human samples [25].

### Predictors of circulating irisin concentration

A considerable number of anthropometric, ergometric, and metabolic predictors of circulating irisin concentration has been proposed [10,17,18,26]. Some of the results are contradictory, most likely due to large differences in the studied populations. In our subjects, who were representative of healthy participants in preventive exercise (and, thus, of the target population likely to have possible irisin effects), only age and total cholesterol could be identified as independent predictors of baseline irisin concentrations, whereas measures of adiposity, physical performance, and glucose homeostasis were not.

To date, potential predictors of training-induced changes in circulating irisin have not been formally investigated. However, an influence of age and adiposity has been assumed [9] (including reply). These factors and gender and baseline irisin concentration could not be confirmed in our data.

### Limitations

In the present study, we assessed only the concentration of circulating irisin and the expression of FNDC5 in skeletal muscle has not been studied. Despite the roughly proportional changes on the circulating protein and mRNA levels reported by Boström *et al*. [1], this difference has to be considered when comparing our results with those of purely mRNA-based analyses [9]. However, only circulating irisin, but not tissue bound FNDC5 mRNA or protein, may cause effects in remote adipose tissue.

A time range of 2 to 7 days was allowed between the final bout of the training exercise phase and the final test of each subject (median: 4 days). This time lag, which is longer than in the original study by Boström *et al*. (48 hours [1]), was chosen because it is clearly longer than the acute effect of exercise for major health-related parameters [27,28], but still avoids de-training effects [29-31]. Although the time course of eventual exercise-induced changes in circulating irisin has not been studied to date, no association between the aforementioned time lag and training effects on circulating irisin became apparent in the training groups in the present study (*P* = 0.656; r = 0.06). Therefore, it is unlikely that a chronic training effect with unusually rapid regression was missed; however, this cannot be excluded completely.

## Conclusions

A general induction of irisin by regular exercise training seems unlikely. This limits the putative role of irisin for training-induced improvements of metabolic health. An induction of irisin in a limited subset of people (elderly or obese) has been reported previously [9] however, age, sex, BMI and baseline irisin value could not be substantiated as predictors in our data.

## Abbreviations

AET: Aerobic endurance training; ANOVA: Analysis of variance; BMI: Body mass index; bpm: Beats per minute; ELISA: Enzyme-linked immunosorbent assay; PGC: Peroxisome proliferator-activated receptor gamma coactivator; SET: Strength endurance training.

## Competing interests

The authors declare that they have no competing interests.

## Authors’ contributions

AH contributed to study conception and design, performed the statistical analysis, and was involved in drafting the manuscript. MW contributed substantially to the conduct of the training study, and participated in drafting the manuscript. AS contributed substantially to the conduct of the training study, participated in drafting the manuscript, and gave final approval for the submitted version. JK contributed substantially to the conduct of the training study, and participated in drafting the manuscript. AM was involved in the design of the study, contributed to the conduct of the training study, and participated in drafting the manuscript. SR carried out the ELISA measurements, and participated in drafting the manuscript. LK contributed to the ELISA measurements and analysis of data, and participated in drafting the manuscript. TM was involved in the concept and design of the study, contributed to data analysis and interpretation, and participated in drafting the manuscript. All authors read and approved the final manuscript.

## Pre-publication history

The pre-publication history for this paper can be accessed here:

http://www.biomedcentral.com/1741-7015/11/235/prepub
